# Environmental and occupational respiratory diseases – 1056. Enhancement of sensitivity for human airway epithelial cells to house dust mites by respiratory syncytial virus infection

**DOI:** 10.1186/1939-4551-6-S1-P54

**Published:** 2013-04-23

**Authors:** Woo-Sung Chang, Jo-Young Son, Eun-Jin Kim, Yeon-Mi Lim, Jung-Won Park, Joo-Shil Lee

**Affiliations:** 1 Center for Immunology and Pathology, Korea National Institute of Health, Korea Center for Disease Control and Prevention, South Korea; 2 Department of Internal Medicine and Institute of Allergy, Yonsei University College of Medicine, South Korea

## Background

Airway is one of the main routes for allergen exposure and entering airborne allergens through the airway can initiate allergic responses, resulting in development of allergic asthma. There are various environmental factors that also influence to the airway-mediated immune responses. Among the factors, respiratory syncytial virus (RSV) enables the condition of the airway to change because RSV infection is common in early life and induce the bronchiolitis. In addition, several studies have been reported that non-immune cells such as airway epithelial cells play a pivotal role in the initiation of allergic responses. The aim of our study is to determine the association between the sensitization of common airborne allergens and RSV infection in airway epithelial cells.

## Methods

We cultured A549 human epithelial cell line in serum-free media for a day and RSV was inoculated into the cells. After the infection for an hour, low or high dose of *Dermatophagoides farinae* (*Der f*) or *Dermatophagoides pteronyssinus* (*Der p*) allergen was treated into media and 24hr later we assessed the concentration of proinflammatory cytokines in media and their mRNA levels.

## Results

IL-8 levels were highly detected in both RSV-infected cells only and allergen-treated cells only group. In low dose allergen-treated groups, particularly, IL-8 concentration in media was significantly higher in RSV-infected group than no-infected group. Other proinflammatory and allergy-related cytokines such as IL-6, TNF-a, and TSLP in RSV and allergen-treated groups showed no difference compared with control groups. The other house dust mite allergen, *Der p* also showed similar results with *Der f*, increasing IL-8 level in RSV-infected with low-dose allergen treated group.

## Conclusions

Our findings showed that IL-8 production in low dose of *Der f*-sensitized human airway epithelial cells was highly enhanced by RSV infection. We therefore implied that RSV infection may allow the airway epithelial cells to be more sensitive against airborne allergens, leading to augmentation of susceptibility for asthma.

